# Preservation Effect of Protein-Based Composite Coating Solution from Highland Barley Distillers’ Grains on Crown Pears

**DOI:** 10.3390/polym17172291

**Published:** 2025-08-24

**Authors:** Qian Lv, Jie Zhao, Yiquan Zhang

**Affiliations:** College of Agriculture and Animal Husbandry, Qinghai University, Xining 810016, China; 15297018607@163.com (Q.L.); 15202531350@163.com (J.Z.)

**Keywords:** highland barley distillers’ grains, gliadin, response surface methodology, crown pears, edible coatings, fruit preservation

## Abstract

Crown pears exhibit high susceptibility to rot and rapid deterioration, resulting in quality degradation and fruit softening. Edible coatings serve as an effective preservation technology to extend their shelf life. In this study, a composite coating solution was formulated using vitamin C (Vc), glycerol, ethanol, and gliadin derived from highland barley distillers’ grains. The coating formulation was optimized via single-factor experiments and Box–Behnken response surface methodology, with crown pears’ weight loss as the evaluation metric. The optimal composition comprised 19.86 mg/mL gliadin, 1.47% (*v/v*) glycerol, 2.49% (*w/v*) Vc, and 80.20% (*v/v*) ethanol, achieving a minimized weight loss of (3.30 ± 0.01)%. Treated pears coated with this optimized formulation were stored for 28 days. Preservation efficacy was evaluated through measurements of weight loss, decay rate, total number of colonies, firmness, titratable acid content, and polyphenol oxidase activity. Compared to the uncoated control group, the composite coating treatment significantly mitigated the decline in firmness, weight loss, and titratable acid content of crown pears. Furthermore, it effectively suppressed the increase in polyphenol oxidase (PPO) activity, decay rate, and total number of colonies, thereby extending the shelf life of the fruit.

## 1. Introduction

Crown pear (Pyrus bretschneideri Rehd) is a nutrient-rich fruit valued for its high polysaccharide content and the presence of malic acid, calcium, iron, and various vitamins. The fruit exhibits a crisp, tender, and juicy texture and is renowned for its throat-moistening and cough-alleviating effects [1,2]. However, pears are highly perishable postharvest, being susceptible to rapid spoilage, quality degradation, and flesh softening, which constitute the primary factors compromising their appearance, texture, flavor, and aroma [3]. Although studies have documented quality changes during crown pear storage to extend shelf life [4], reports on dedicated preservation techniques remain scarce. Consequently, developing novel preservation technologies for crown pears is of significant importance.

Edible coatings represent a class of protective solutions applied to food surfaces that form functional films upon cooling, offering a natural, cost-effective preservation alternative [5,6,7]. These coatings function as barriers against microorganisms, water vapor, and gases, thereby slowing postharvest metabolic processes and extending fruit shelf life [8]. Typically composed of polysaccharides, proteins, and/or lipids, they form semi-permeable membranes on the fruit surface. Protein-based films are particularly advantageous due to their excellent barrier properties against water vapor and gases, alongside their inherent safety, biocompatibility, and suitability for fruits and vegetables [9]. Current protein sources explored for fruit coatings include bee pollen protein, wheat gluten, and maize zein, with zein being the most extensively characterized. For instance, a coating comprising 5% zein and 10% gelatin delays mango ripening, maintains quality attributes, and extends shelf life during storage [10]. Similarly, Baraiya et al. [11] demonstrated that coating blackberries (*Syzygium cumini* L. var. Paras) with antioxidant-rich zein enhanced their postharvest quality and storage duration. However, the preparation of gliadin-based preservation solutions from highland barley distillers’ grains and their application to fruit preservation remain largely unexplored.

Highland barley (*Hordeum vulgare* L. var. nudum Hook. f) contains elevated levels of functional components such as protein, dietary fiber, and vitamins compared to staple grains like rice, wheat, and maize. Its protein content ranges from 6.35% to 23.40% (mean: 12.43%), comprising globulin, albumin, gliadin, and glutenin [12,13]. Highland barley distillers’ grains, a byproduct of highland barley distillers’ grains, are notably protein-rich, reaching 23.57% protein content [14]. Gliadin constitutes a significant portion of this protein, accounting for 21.04% of the protein fraction [15]. As a cereal storage protein, gliadin exhibits inherent hydrophobicity due to its high content of nonpolar amino acids such as leucine and proline, rendering it capable of forming films with excellent water vapor barrier properties. However, films derived solely from highland barley distillers’ grain gliadin exhibit limitations, including high brittleness, poor mechanical strength, and significant sensitivity to environmental temperature and humidity (e.g., swelling in high-humidity conditions), resulting in poor stability. To enhance performance for practical application, modifications via physical or chemical methods or blending with functional additives are required. For instance, incorporating glycerol as a plasticizer can improve film flexibility and mechanical properties by reducing intermolecular forces between polymeric chains and increasing susceptibility to water vapor penetration [16,17,18].

Ascorbic acid (AsA, Vitamin C), a crucial water-soluble antioxidant widely utilized in food and agricultural systems, inhibits enzyme-catalyzed browning in postharvest fruits and helps prevent the loss of endogenous nutrients during shelf life [19,20]. Furthermore, the moist surfaces and nutrient-rich nature of harvested fruits provide ideal substrates for microbial growth. Thus, to mitigate microbial spoilage, composite coatings should incorporate ethanol-based antimicrobial agents. For example, composite treatments utilizing ethanol (e.g., 20%) and Vc (e.g., 1%) have proven effective in maintaining the postharvest appearance quality of apples [21].

Therefore, this study utilizes gliadin extracted from highland barley distillers’ grains combined with Vc, glycerol, and ethanol to formulate a composite coating solution. Given its simplicity and cost-effectiveness, dip coating was employed to apply the composite to crown pears. Subsequently, using weight loss as the primary indicator, the optimal formulation of the composite coating solution was determined employing response surface methodology optimization. For the first time, this research systematically evaluates the effects of highland barley distillers’ grain gliadin-based coating on key quality parameters of crown pears—including weight loss, decay rate, hardness, titratable acid content (TA), polyphenol oxidase (PPO) activity, and total number of bacterial colonies—over a 28-day storage period. This work establishes a foundation for the comprehensive utilization and valorization of highland barley distillers’ grains. 

## 2. Materials and Methods

### 2.1. Materials

Highland barley distillers’ grains were obtained from the Tianyoude Barley Wine Co., Ltd. (Haidong, China); crown pears were obtained from Jinzhou City (Hebei, China). The gliadin of highland barley distiller’s grains was prepared by the laboratory of the research group; 95% ethanol, phenolphthalein indicator, glycerol, Vc, glacial acetic acid, hydroquinone, and sodium hydroxide were of analytical grade were acquired from Qinghai Rhein Bio-Technology Co., Ltd. (Xining, China).

### 2.2. Extraction of Gliadin from Highland Barley Distillers’ Grains

Following a protocol adapted from Guo et al. [22], highland barley distillers’ grains were dried in a drying oven at 50 ± 0.5 °C for precisely 30 min. Dried highland barley distillers’ grains were ground in a high-speed grinding mill and passed through a 60-mesh sieve. Subsequently, 200 g of the powder sample was dissolved in 600 mL of an 80% ethanol solution. And samples were magnetically agitated at 500 rpm for exactly 20 min under 25 ± 2 °C using a magnetic stirrer. Ultrasonication was performed at an ultrasonic power of 400 W and a temperature of 50 °C for 120 min. Post-sonication samples underwent centrifugation (8000× *g*, 20 min, 4 °C) in a high-speed refrigerated centrifuge. The supernatant was dialyzed against ultrapure water in the dark (12 kDa, 48 h, 4 °C) to remove low-molecular-weight impurities. The retentate was concentrated by rotary evaporation under reduced pressure (40 °C, 60 rpm) to eliminate residual ethanol and water. Subsequently, the samples were freeze-dried for 48 h to constant weight using a vacuum freeze dryer with condenser temperature maintained at −53 °C.

### 2.3. Preparation of Protein-Based Composite Coating Liquid from Highland Barley Distillers’ Grains

Following the method described by Zu et al. [23] with modifications: ① Gliadin (20 mg/mL): 5.00 ± 0.01 g gliadin dissolved in 70% ethanol. ② Vc (2% *w/v*): 5.00 ± 0.01 g Vc in DI water. ③ Ethanol (75% *v/v*): 100 mL anhydrous ethanol diluted to final volume. ④ Glycerol (1.5% *v/v*): 10 mL glycerol in DI water. Composite solutions were mixed thoroughly by magnetic stirring (500 rpm, 25 ± 0.5 °C, 20 min). Crown pears exhibiting uniform ripeness, color, and absence of defects were selected. Fruits were randomized into the experimental group (EG; *n* = 15; 3 replicates × 5 fruits) and control group (BG; *n* = 15; untreated). EG samples were immersed in coating solution (25 ± 0.5 °C) for 10.0 ± 0.5 s, drained for 120 ± 5 s on sterile racks, and stored at 25 ± 0.5 °C/85 ± 3% RH for 28 d. Weight loss was determined at 7-day intervals.

### 2.4. Single-Factor Experiment

The concentration of the Vc solution was fixed at 2%, the glycerol solution at 1.5%, and the ethanol solution at 75%. The study investigated the effect of different gliadin concentrations (10 mg/mL, 15 mg/mL, 20 mg/mL, 25 mg/mL, and 30 mg/mL) on the weight loss of crown pears. The concentration of gliadin solution was fixed at 20 mg/mL, glycerol solution concentration at 1.5%, and ethanol solution concentration at 75%. The effects of different Vc solution concentrations (1%, 1.5%, 2%, 2.5%, and 3%) on the weight loss of crown pears were investigated. The concentration of gliadin solution was fixed at 20 mg/mL, the concentration of Vc solution was 2%, and the concentration of ethanol solution was 75%. The effects of different glycerol solution concentrations (0.5%, 1%, 1.5%, 2%, and 2.5%) on the weight loss of crown pears were investigated. With the gliadin solution concentration fixed at 20 mg/mL, the Vc solution concentration at 2%, and the glycerol solution concentration at 1.5%, the effects of different ethanol solution concentrations (65%, 70%, 75%, 80%, and 85%) on the weight loss of crown pears were investigated.

### 2.5. Response Surface Experiment

Based on Box–Behnken experimental design methodology and single-factor experiment results, we selected four parameters significantly influencing the weight loss of crown pears: gliadin concentration, glycerol concentration, Vc concentration, and ethanol concentration. Each factor was assigned three coded levels (−1, 0, +1). Analysis of 29 experimental runs via response surface methodology was performed using Design-Expert 10.0.1 software to identify the optimal gliadin-based composite coating formulation derived from highland barley distillers’ grains.

### 2.6. Measurement of Indicators

#### 2.6.1. Weight Loss

The weight loss was determined according to AOAC [24], each determination was run in triplicate, as shown in Equation (1):

(1) $$WL=M_{a}-M_{b}/M_{a}\times 100$$

where *WL* is the weight loss (%), *M_a_* is the quality after freshness treatment (g), and *M_b_* is the mass after freshness treatment and storage for a while (g).

#### 2.6.2. Decay Rate

Three parallel tests were conducted on the EG and BG groups. The decay rate was determined according to Equation (2) as follows:

(2) DR=n/N×100

where *DR* is the decay rate (%), *n* is the number of decayed fruits, and *N* is the total number of fruits investigated.

#### 2.6.3. Hardness

The hardness was determined according to Khodaei et al. [25]; measurements were carried out using a TA-XT Plus Texture Analyzer (Stable Micro Systems, Godalming, UK). A 3 mm diameter, cylindrical, flat-bottomed probe was used. The penetration speed was set at 1 mm/s, with the penetration depth at 6 mm, and the results were expressed as peak forces in newtons (N). Each pear was measured at least three times in different locations, and the results were averaged.

#### 2.6.4. Titratable Acid Content

Crown pear samples of control and experimental groups were weighed at 2.5 g each and ground into 25 mL volumetric flasks; distilled water was added and samples were fixed to the scale, mixed well, and filtered. Take 10 mL of filtrate in a beaker, add 2 drops of phenolphthalein indicator, titrate the solution with 0.1 mol/L NaOH to a light red color, and do not change color within 30 s. Record the amount of NaOH solution. Each determination was run in triplicate. The titratable acid content was determined according to Equation (3) as follows:

(3) $$TA=V\times C\times \left(V1-V0\right)\times f/\left(VS\times m\right)\times 100$$

where *TA* is the titratable acid content (%), *V* is the total volume of the sample, 25 mL, *C* is the concentration of NaOH titrant, 0.1 mol/L, *V*1 is the volume of NaOH solution consumed by the titration of the sample solution (mL), *V*0 is the volume of NaOH solution consumed by the titration of the distilled water (mL), *f* is the conversion factor, 0.075 g/mmol, *VS* is the volume of the filtrate for the titration, 10 mL, and *m* is the mass of the sample, 2.5 g.

#### 2.6.5. Polyphenol Oxidase Activity

PPO activity was determined using the catechol method. The increase in absorbance at 420 nm due to PPO activity was monitored at one-minute intervals using a UV-2600 spectrophotometer (Shanghai Yidian Analytical Instruments Co., Ltd., Shanghai, China). Each treatment group was measured for five minutes to obtain six sets of data, which were repeated three times. The results were considered one unit of enzyme activity (U) per gram of fruit sample (fresh weight) for each 1 min increase in absorbance. The unit of PPO activity is U/g FW.

#### 2.6.6. Total Number of Colonies

For the calculation, the plate counting method was employed, as stated in GB4789.2-2022. Each determination was run in triplicate.

#### 2.6.7. Statistical Analysis

Data are reported as mean ± standard deviation (Mean ± SD) in triplicate. Statistical analysis was performed using SPSS 27 software (SPSS Inc., Chicago, IL, USA). Depending on the specific experimental design and data characteristics, data were analyzed using one-way analysis of variance (ANOVA) and independent sample *t*-tests. The criterion for statistical significance was set at *p* < 0.05. Data visualization was performed using Design-Expert 10.0.1 and Origin 2021 software.

## 3. Results and Discussion

### 3.1. Analysis of the Single-Factor Results

#### 3.1.1. Effect of Different Gliadin Concentrations on the Weight Loss of Crown Pears

**Figure 1:**
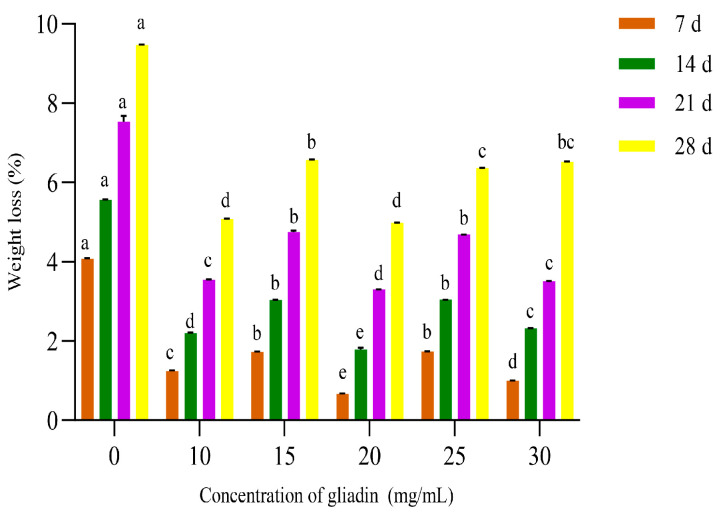
Influence of different gliadin concentrations on the weight loss of crown pears. Note: Different lowercase letters indicate significant differences within groups, *p* < 0.05 (Figure 2, Figure 3 and Figure 4 are the same as above).

As shown in Figure 1, under identical gliadin concentrations, the weight loss across all groups increased with prolonged storage duration. This phenomenon is primarily attributed to moisture evaporation from fruit tissues during extended aerial exposure, leading to dehydration and consequent weight reduction [26]. When storage time was held constant, experimental groups coated with varying gliadin concentrations exhibited significantly lower weight loss compared to the uncoated control group (*p* < 0.05). Furthermore, the weight loss of crown pears demonstrated a distinct nonlinear response to incremental changes in gliadin concentration. At a gliadin concentration of 20 mg/mL, weight loss reached its minimum across all storage intervals. This optimal efficacy is attributed to the inherent hydrophobicity of gliadin, enabling the formation of a dense molecular barrier on the fruit surface that effectively impedes water vapor diffusion [27].

However, exceeding the 20 mg/mL threshold induced protein aggregation driven by intensified intermolecular hydrophobic interactions and hydrogen bonding networks. This structural rearrangement resulted in diminished packing density within the coating, consequently compromising its barrier efficacy against moisture permeation. Leveraging this nonlinear response pattern, representative concentrations of 15, 20, and 25 mg/mL were selected for subsequent experimental framework development.

#### 3.1.2. Effect of Different Glycerol Concentrations on the Weight Loss of Crown Pears

As shown in Figure 3, under equivalent storage durations, experimental groups treated with distinct glycerol concentrations exhibited significantly reduced weight loss compared to the uncoated control (*p* < 0.05). The weight loss of crown pears demonstrated a pronounced nonlinear response to incremental changes in glycerol concentration. The minimal weight loss was achieved at a glycerol concentration of 2.0%. This optimal efficacy likely stems from glycerol’s exceptional wettability. The hydrophilic hydroxyl groups intrinsic to glycerol molecules engage surrounding water vapor via hydrogen bonding, significantly impeding trans-epidermal water migration [28].

Concentrations exceeding 2.0% glycerol cause the membrane to become overly swollen, forming localized droplets and significantly increasing water activity [29]. This activates microbial β-glucosidase, increasing the secretion intensity of extracellular enzymes and accelerating the degradation of pectin. This results in structural water loss [30]. Therefore, representative concentrations of 1.5%, 2.0%, and 2.5% were selected for subsequent experimental framework development based on this nonlinear response profile.

**Figure 2:**
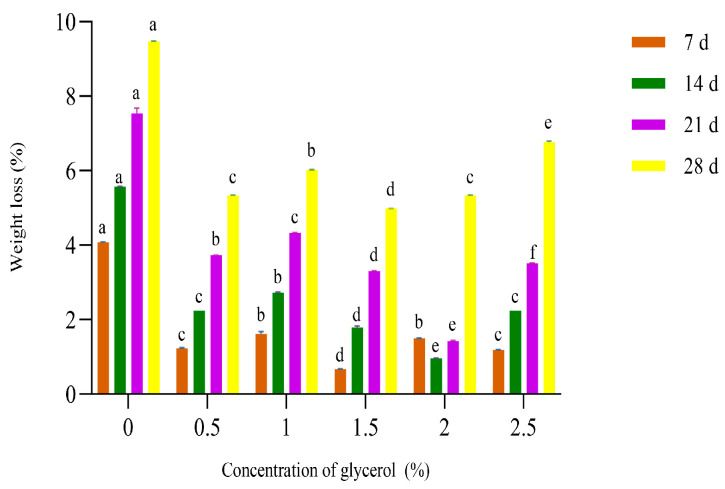
Influence of different glycerol concentrations on the weight loss of crown pears.

#### 3.1.3. Effect of Different V_C_ Concentrations on the Weight Loss of Crown Pears

**Figure 3:**
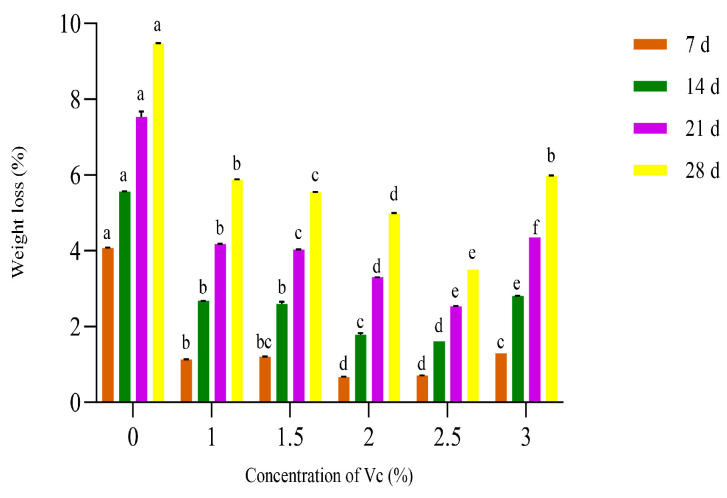
Influence of different vitamin C concentrations on the weight loss of crown pears.

As shown in Figure 3, when the V_C_ concentration was the same, the weight loss of each group increased with the increase in storage time, and when the storage time was the same, the weight loss of each experimental group with different V_C_ concentrations was significantly reduced (*p* < 0.05) compared with the uncoated control group, and the weight loss of crown pears showed a significant nonlinear response characteristic with the change in V_C_ concentration gradient. The crown pear system presented the lowest weight loss when the V_C_ concentration was 2.0%. This was mainly attributed to the many hydroxyl groups of V_C_ [31]. Therefore, V_C_ is strongly hydrophilic and can capture environmental water molecules, effectively keeping the internal environment of crown pears in a humid state, thus reducing weight loss due to water evaporation. Ali et al. [32] showed that exogenous application of ascorbic acid to lychee fruit inhibited postharvest senescence and weight loss.

Vc concentrations above 2.5% induced a significant increase in weight loss, demonstrating that supraoptimal Vc levels accelerate fruit senescence. Such senescence impairs cellular integrity and reduces overall fruit quality. Based on the above nonlinear response relationship, three characteristic concentration gradients of 1.5%, 2.0%, and 2.5% were selected in this study for subsequent experimental system construction.

#### 3.1.4. Effect of Different Ethanol Concentrations on the Weight Loss of Crown Pears

**Figure 4:**
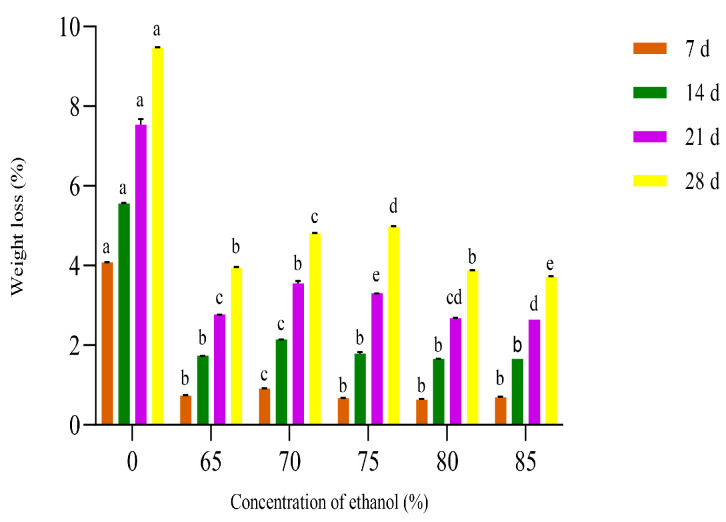
Influence of different ethanol concentrations on the weight loss of crown pears.

As shown in Figure 4, the weight loss of each experimental group increased with storage time at the same ethanol concentration. This phenomenon was mainly due to the fruit being exposed to air for a long time, which led to water loss and decreased weight [26]. With the same storage time, the weight loss of each experimental group with a different ethanol concentration was significantly lower (*p* < 0.05) than that of the uncoated control group. The weight loss of crown pears showed a significant nonlinear response characteristic with the ethanol concentration gradient. When the ethanol concentration reaches 85%, the weight loss of the crown pear system decreases to its lowest value due to ethanol’s strong penetration of cells, which can lead to the permanent denaturation of microbial cell proteins, reduce nutrient consumption in crown pears, slow tissue aging, and extend storage time [33]. Zhang et al. [34] showed that appropriate ethanol treatment can delay postharvest senescence of table grapes, which is conducive to reducing fruit quality loss.

Based on this nonlinear response law, three characteristic concentrations of 75%, 80%, and 85% were selected in this study to construct the optimization model for validation experiments.

### 3.2. Optimization of Response Surface Experiment

**Figure 5:**
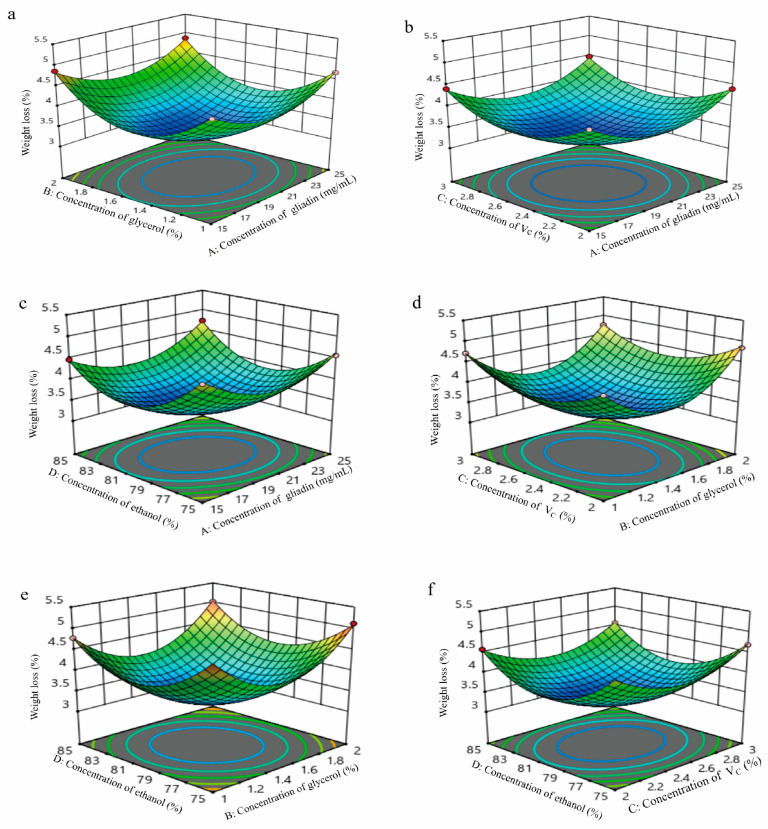
Influence of the interaction of factors on the weight loss of crown pears. (**a**) AB; (**b**) AC; (**c**) AD; (**d**) BC; (**e**) BD; (**f**) CD.

The design scheme and results are shown in Table 1 and Table 2, the ANOVA results are shown in Table 3, and the results of the response surface and contour plots obtained by using Design Expert 10.0.1 software are shown in Figure 5a–f.

A multiple regression model was fitted to the data in Table 2, yielding the following linear regression equation: Y = 3.304 + 0.0258333A + 0.0925B + 0.0141667C − 0.0591667D − 0.02AB + 0.005AC + 0.1075AD − 0.0625BC + 0.04BD − 0.005CD + 0.555083A^2^ + 0.890083B^2^ + 0.542583C^2^ + 0.782583D^2^. As shown in Table 3, the model’s F-value is 456.10, which is highly significant (*p* < 0.0001). The loss-of-fit term is not significant (*p* = 0.2238 > 0.05), indicating that the model fits well and the experimental value is close to the predicted value. Therefore, it can be used to analyze and predict crown pears’ weight loss. The weight loss of crown pears can be analyzed and predicted. The coefficient of determination, R^2^, is 0.9978, and the modified R^2^ is 0.9956. These values indicate that 99.56% of the data can be explained by the model, meaning the equation is highly reliable.

As seen from the *p*-value, primary terms B and D have a highly significant effect on crown pear weight loss (*p* < 0.01), while A has a significant effect (*p* < 0.05). Its secondary term interactions, AD and BC, also have a highly significant effect (*p* < 0.01), BD has a significant effect (*p* < 0.05), and the secondary terms A^2^, B^2^, C^2^, and D^2^ have a highly significant effect (*p* < 0.01). Combined with the *F*-value, it can be seen that the interaction term AD has the greatest influence on the weight loss of crown pears, followed by BC and BD. The F value shows that the factors influencing the weight loss of crown pears are B (glycerol concentration) > D (ethanol concentration) > A (gliadin concentration) > C (V_C_ concentration).

Response and contour surface plots were obtained using Design-Expert 10.0.1 software to visually analyze the influence pattern of each factor on the weight loss of crown pears.

### 3.3. Optimization and Validation Tests

The regression model predicted optimum coating parameters as gliadin 19.858 mg/mL, glycerol 1.473% (*w/w*), Vc 2.492% (*w/v*), and ethanol 80.205% (*v/v*). After instrument-resolution-informed adjustment to practical precision (gliadin 19.86 mg/mL, glycerol 1.47% (*w/w*), Vc 2.49% (*w/v*), ethanol 80.20% (*v/v*)), validation trials demonstrated the weight loss of crown pears (3.30 ± 0.02)%. This result shows negligible deviation from the predicted 3.30%, confirming model robustness (relative error: 0.3%).

### 3.4. Measurement Results of Indicators

#### 3.4.1. Changes in Weight Loss of Crown Pears

**Figure 6:**
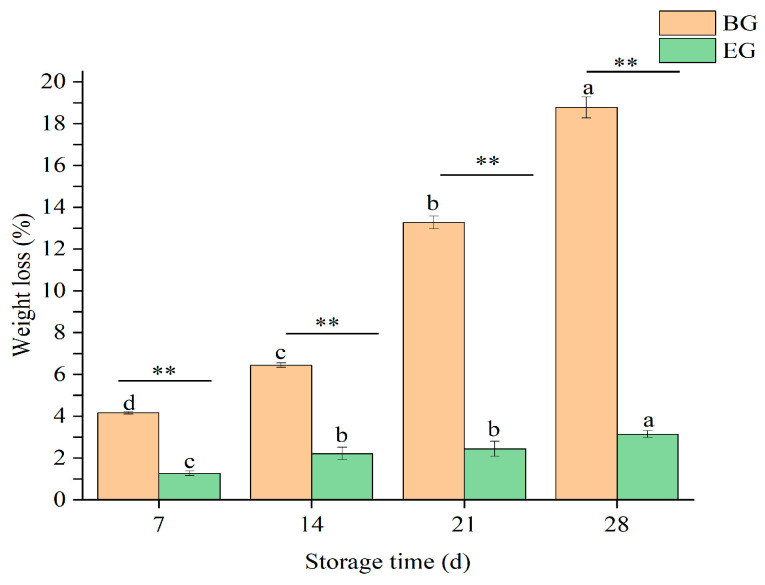
Influence of protein-based composite coating solution from highland barley distillers’ grains on the weight loss of crown pears. Note: BG: blank group, EG: experimental group, different lowercase letters indicate significant differences within groups, *p* < 0.05. *p* < 0.01 indicates highly significant differences between groups and is denoted by ** (Figures 9–11 are the same as above).

As shown in Figure 6, crown pears exhibited a significant increase in weight loss across all sample groups during the 28-day storage period (*p* < 0.05). The uncoated control group displayed a weight loss of (18.77 ± 0.50)%, whereas pears treated with the composite protein-based coating solution showed substantially reduced weight loss (3.13 ± 0.16)%. This represents a highly significant reduction in weight loss relative to controls (*p* < 0.01). This phenomenon may be attributed to the formation of a multifunctional, semipermeable film on the surface of the pears by the protein-based composite coating solution. Edible coatings can reduce quality loss by minimizing water loss in fruits that have not undergone excessive processing [35,36]. In terms of water evaporation, the protective film acts as a barrier against gas and water leakage. This effectively reduces the rate of water vapor exchange between the fruit and the external environment, thereby reducing water dissipation. In terms of respiration, the film blocks access to some oxygen, which inhibits the fruit’s respiratory metabolism. Odilio et al. [27] coated apple slices using a formulation of 4.0% zein, 0.25% oleic acid as a plasticizer, and 70% ethanol. The results showed that the coating reduced the rate of weight loss in apples, which agrees with the results of this study.

#### 3.4.2. Changes in Decay Rate of Crown Pears

**Figure 7:**
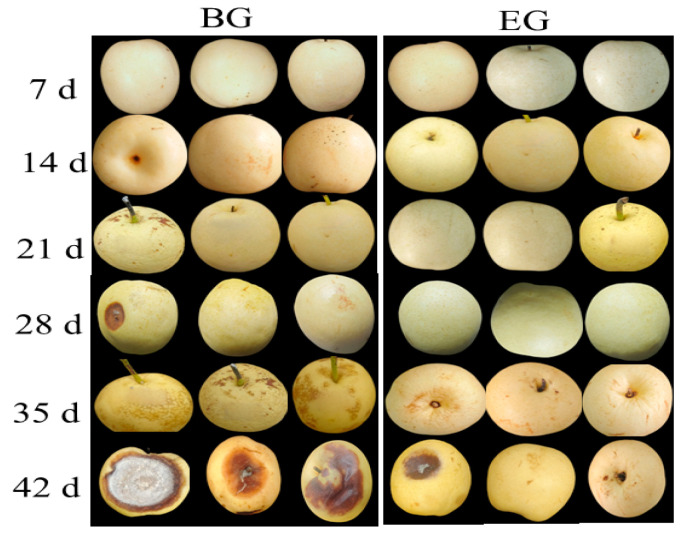
Influence of protein-based composite coating solution from highland barley distillers’ grains on the appearance of crown pears.

Increased fruit dryness and weight loss were related to water loss and exhibited a parallel trend with the decay rate [37]. As shown in Figure 7, during storage, the decay rate of crown pears increased gradually over time. In the middle and late stages of storage, the blank control group without treatment began to rot. As shown in Table 4, after 28 days, 33.3% of the blank group had spoiled, while none of the test group had spoiled. By the 42nd day, the decay rate of the control group had reached 100%. After 42 days of storage, 100% of the items in the blank group had spoiled, while only 33% of the items in the test group had spoiled. This indicates that the composite film preservation solution can effectively delay the increase in the decay rate of crown pears. Fruit deterioration during storage is mainly caused by water loss and wilting. Edible coatings prevent excessive fruit transpiration by providing a barrier, which reduces the fruit’s decay rate [38]. Our results were confirmed by Gol et al. [10], who found that mangoes treated with a zein coating retained their quality and had a lower decay rate compared to uncoated samples.

**Table 4:** The effect of protein-based composite coating preservation solution on the decay rate of crown pears.

Storage Time (d)	Blank Group Decay Rate (%)	Experimental Group Decay Rate (%)
7	0	0
14	0	0
21	0	0
28	33.30%	0
35	66.60%	0
42	100%	33.00%

#### 3.4.3. Changes in Hardness of Crown Pears 

**Figure 8:**
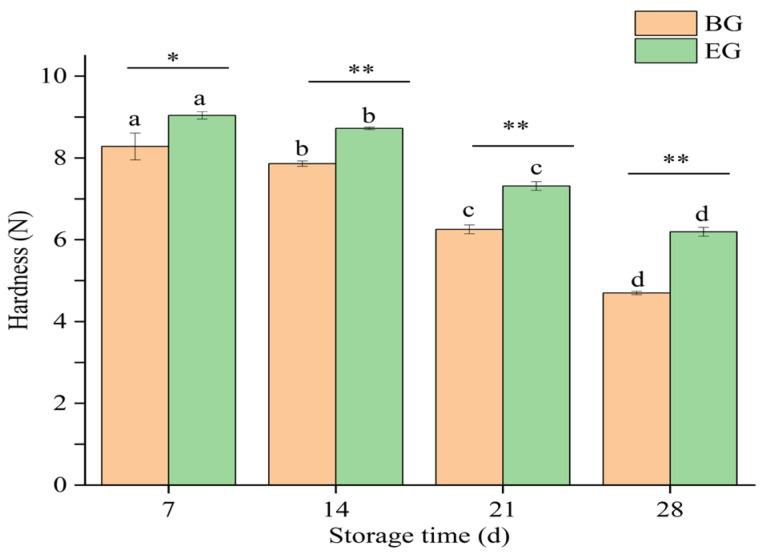
Influence of protein-based composite coating solution from highland barley distillers’ grains on the hardness of crown pears. Note: BG: blank group, EG: experimental group, different lowercase letters indicate significant differences within groups, *p* < 0.05. *p* < 0.01 indicates highly significant differences between groups and is denoted by **, *p* < 0.05 indicates significant differences between groups and is denoted by *.

Hardness is an important factor influencing consumer acceptance of fresh fruits [39], and their changes reflected the alterations in the internal structure and physiological state of the fruit. As shown in Figure 8, the hardness of crown pears in both groups decreased significantly with the prolongation of storage time (*p* < 0.05); from the early stage of storage to the 28nd day, the hardness of crown pears in the blank control group decreased from (8.28 ± 0.32) N to (4.69 ± 0.04) N, and that of crown pears in the composite coating group decreased from (9.04 ± 0.08) N to (6.19 ± 0.10) N. The hardness of the crown pears treated with the composite coating was consistently and significantly higher than that of the control group (*p* < 0.01). The hardness of crown pears in the composite coating group was consistently higher than that of the control group. The hardness of the treated pears was always higher than that of the control group, and the composite coating group had the slowest decreasing trend. On the one hand, this was because the softening of the fruit was related to water loss, and the composite coating film formed a barrier on the surface of the fruit to prevent the penetration of water [40], which reduced the loss of water, inhibited the softening of the fruit, and maintained a relatively high hardness. On the other hand, the decrease in fruit firmness is attributed to the increase in endogenous autolysin activity, which leads to cell wall degradation [41].

When the activities of cell wall-degrading enzymes such as polygalacturonase, pectin methylesterase, and β-galactosidase are increased, which in turn reduces intercellular adhesion and the mechanical strength of the cell wall, a decrease in flesh firmness occurs during fruit ripening [42]. However, these enzymes require oxygen to function, and the composite coating material acts as a barrier to prevent oxygen penetration [40] and reduces cell wall degradation, thus slowing down the softening process of crown pears.

#### 3.4.4. Changes in Titratable Acid Content of Crown Pears 

**Figure 9:**
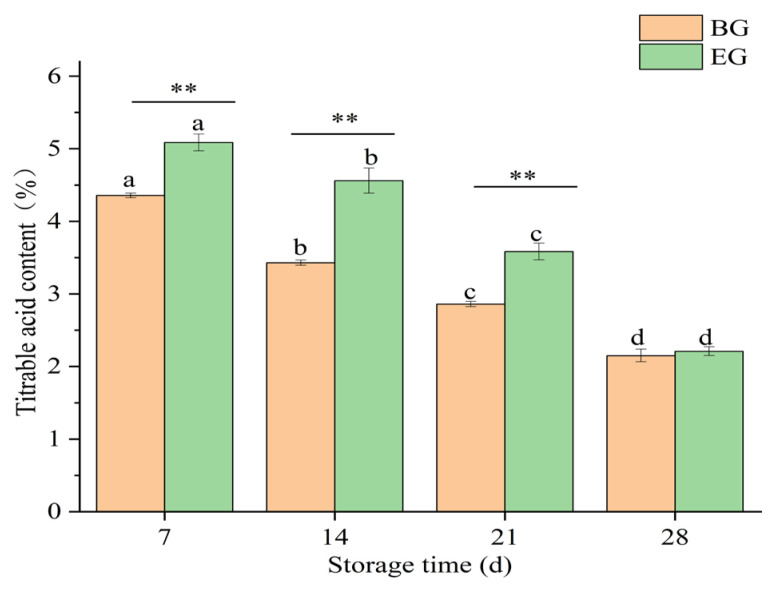
Influence of protein-based composite coating solution from highland barley distillers’ grains on the titratable acid content of crown pears.

Titratable acid content is an important indicator of fruit quality and flavor. Its changes reflect dynamic physiological and biochemical processes within the fruit. As shown in Figure 9, the titratable acid content of crown pears decreased significantly during storage (*p* < 0.05). In the blank control group, it decreased from 4.35% on day 7 to 2.86% on day 21. In the experimental group, it decreased from 5.08% on day 7 to 3.58% on day 21. The decrease in crown pears’ acidity may be related to the conversion of organic acids to sugars or their derivatives, or their utilization during respiration and storage [43,44].

However, the titratable acid content in the experimental group was significantly higher than in the blank control group (*p* < 0.01). The results indicated that the composite-coated freshness preservation solution effectively delayed the reduction of organic acids and prolonged the shelf life of crown pears. Studies have shown that edible coatings may slow the rate of respiration, thereby delaying the utilization of organic acids in fruits [45]. Gol et al. [46] showed that the coating slowed the changes in TA by delaying the aging process in banana fruits. This is consistent with the results of the present study.

#### 3.4.5. Changes in PPO of Crown Pears 

Postharvest browning of fruits is mainly caused by polyphenol oxidase (PPO), as it oxidizes phenolic compounds [47]. When plant tissues are damaged, polyphenols present in various organelles interact with PPO [48], which acts as a key enzyme in causing fruit browning, and the level of its activity directly affects the quality of the fruit.

**Figure 10:**
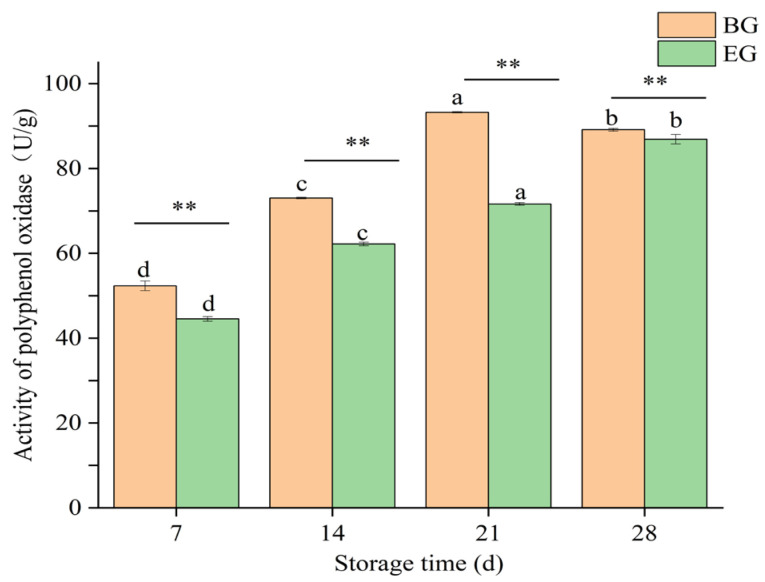
Influence of protein-based composite coating solution from highland barley distillers’ grains on the PPO of crown pears.

As shown in Figure 10, the polyphenol oxidase (PPO) activity of crown pears from the untreated control group showed a dynamic trend of first increasing and then decreasing over time during storage. However, the PPO activity of the experimental group treated with the protein-based composite coating solution was consistently and significantly lower than that of the untreated control group throughout the storage process (*p* < 0.01). On the 28th day of storage, the PPO activity of the control group reached (89.13 ± 0.35) U/g FW, while that of the treated group was only (86.90 ± 1.11) U/g FW. The lower PPO activity of the treated group indicated that the protein-based composite coating solution had a significant inhibitory effect on the PPO activity of crown pears. This inhibitory effect may be due to the anti-browning effect of ascorbic acid on the one hand, and the slowing down of the enzymatic browning reaction of the fruit on the other. For example, Liu et al. [49] used ASA to treat postharvest fresh longan fruit; the results showed that ASA treatment could effectively reduce the weight loss of the fruit and inhibit the browning of the peel. On the one hand, edible coatings provide a barrier against oxygen and water availability by protecting the fruit from enzymatic oxidation of phenolic compounds [50,51].

#### 3.4.6. Changes in Total Number of Colonies of Crown Pears

**Figure 11:**
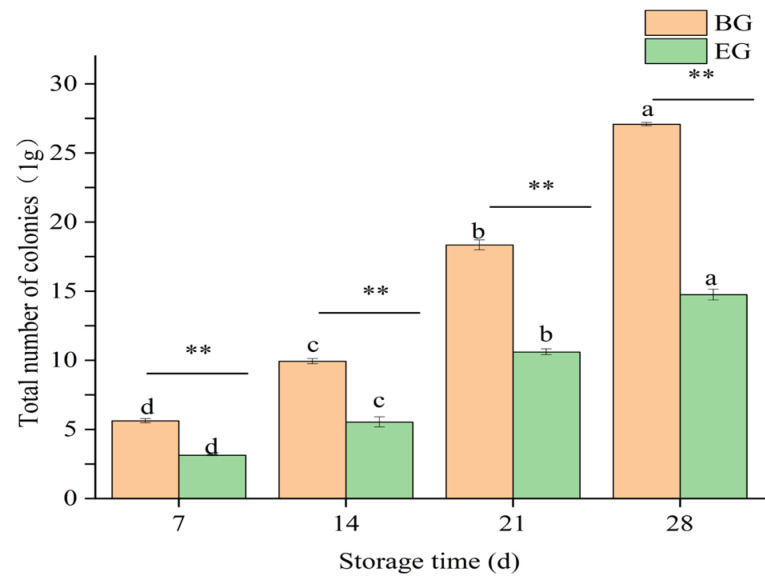
Influence of protein-based composite coating solution from highland barley distillers’ grains on the total number of colonies of crown pears.

The total number of colonies is a key indicator of microbial contamination of crown pears, and its value is positively correlated with the likelihood of spoilage of the samples. As shown in Figure 11, the total number of crown pear colonies in each group increased significantly (*p* < 0.05) during the 28-day storage period. The blank control group had a total of (27.07 ± 0.12) CFU/g after 28 days. However, the total number of colonies in the experimental group treated with a protein-based composite coating solution was (14.75 ± 0.39) CFU/g. This value was significantly lower than that of the blank control group that was not treated (*p* < 0.01). This result indicated that the composite coating solution was effective in inhibiting the growth and multiplication of microorganisms and reducing the number of microorganisms. Baysal et al. [52] carried out a study in which apricots were soaked in natural maize zein at moderate humidity and found that the maize zein coatings inhibited microbial growth, which is in agreement with the results of the present study. Although gliadin has weak antibacterial effects, this experiment combined gliadin with ethanol to achieve broad-spectrum antibacterial properties. Chitosan, although inherently antibacterial, requires dissolution in an acidic solution (such as hydrochloric acid or acetic acid, the most effective acids) and subsequent adjustment to the optimal pH value (5.6) when used in experiments [53]. This process typically takes 1–2 days, and compared with gliadin, it is more complex.

## 4. Conclusions

In this study, we optimized the formulation of the protein-based composite coating solution from highland barley distillers’ grains through single-factor experiments and Box–Behnken response surface methodology. Subsequently, crown pears were coated under the optimal formulation conditions and stored at 25 °C for 28 days. The weight loss, decay rate, total number of bacterial colonies, hardness, titratable acid content, and polyphenol oxidase activity of crown pears were used as the evaluation indexes to investigate the freshness preservation effect of the composite coating solution on crown pears. It was found that the optimal ratio of the gliadin from highland barley distillers’ grains was 19.86 mg/mL, 1.47% glycerol (*v/v*), 2.49% Vc (*w/v*), and 80.20% ethanol (*v/v*), and the weight loss of crowned pears could be reduced to (3.30 ± 0.01)% under this condition. All the indicators of crown pears treated with composite coating solution were better than those of uncoated crown pears, and the composite coating preservation solution obtained by the study has an obvious preservation effect on crown pears, which is of great significance to prolong the storage period of crown pears. Further studies are needed to investigate the specific mechanisms by which gliadin from highland barley distillers’ grains, glycerol, ethanol, and vitamin C affect fruit preservation.

In the future, the composite-coated preservation solution can be utilized on other fruits, vegetables, and meat products to expand its applications in the food industry.

## Figures and Tables

**Table 1:** Level of factors in the response surface analysis experiment design.

Levels	Factors			
	A. Gliadin Concentration/(mg/mL)	B. Glycerol Concentration /%	C. V_C_ Concentration /%	D. Ethanol Concentration /%
−1	15	1	2	75
0	20	1.5	2.5	80
1	25	2	3	85

**Table 2:** Results of the Box–Behnken test.

NO.	A. Gliadin Concentration /(mg/mL)	B. Glycerol Concentration /%	C. Vc Concentration /%	D. Ethanol Concentration /%	WL /%
1	0	1	0	−1	5.12 ± 0.05
2	0	0	0	0	3.30 ± 0.06
3	0	−1	0	1	4.78 ± 0.02
4	−1	0	1	0	4.41 ± 0.02
5	1	0	1	0	4.48 ± 0.06
6	0	−1	−1	0	4.56 ± 0.02
7	−1	0	0	−1	4.74 ± 0.03
8	0	0	−1	1	4.58 ± 0.02
9	0	0	0	0	3.27 ± 0.02
10	1	0	−1	0	4.41 ± 0.03
11	0	0	0	0	3.29 ± 0.06
12	−1	0	0	1	4.47 ± 0.02
13	1	1	0	0	4.87 ± 0.04
14	0	0	0	0	3.32 ± 0.08
15	0	0	1	−1	4.69 ± 0.02
16	0	−1	0	−1	5.04 ± 0.06
17	1	0	0	−1	4.57 ± 0.11
18	0	0	1	1	4.56 ± 0.03
19	1	−1	0	0	4.68 ± 0.02
20	−1	1	0	0	4.86 ± 0.03
21	0	1	0	1	5.02 ± 0.11
22	−1	0	−1	0	4.36 ± 0.06
23	0	1	1	0	4.76 ± 0.08
24	0	1	−1	0	4.85 ± 0.08
25	0	0	0	0	3.34 ± 0.03
26	1	0	0	1	4.73 ± 0.07
27	−1	−1	0	0	4.59 ± 0.06
28	0	0	−1	−1	4.69 ± 0.08
29	0	−1	1	0	4.72 ± 0.05

Note: Values presented are means ± standard deviation of data from triplicate analysis on duplicate trials.

**Table 3:** Analysis results of the regression equation.

Source	Sum of Squared Deviations	Df	Mean Square	*F*-Value	*p*-Value	Significance
Model	8.87	14	0.63	456.10	<0.0001	**
A	8.008 × 10^−3^	1	8.008 × 10^−3^	5.76	0.0308	*
B	0.10	1	0.10	73.89	<0.0001	**
C	2.408 × 10^−3^	1	2.408 × 10^−3^	1.73	0.2091	
D	0.042	1	0.042	30.23	<0.0001	**
AB	1.600 × 10^−3^	1	1.600 × 10^−3^	1.15	0.3014	
AC	1.000 × 10^−4^	1	1.000 × 10^−4^	0.072	0.7924	
AD	0.046	1	0.046	33.27	<0.0001	**
BC	0.016	1	0.016	11.24	0.0047	**
BD	6.400 × 10^−3^	1	6.400 × 10^−3^	4.61	0.0499	*
CD	1.000 × 10^−4^	1	1.000 × 10^−4^	0.072	0.7924	
A^2^	2.00	1	2.00	1438.33	<0.0001	**
B^2^	5.14	1	5.14	3698.32	<0.0001	**
C^2^	1.91	1	1.91	1374.28	<0.0001	**
D^2^	3.97	1	3.97	2858.94	<0.0001	**
Residual	0.019	14	1.390 × 10^−3^			
Lack-of-fit-term	0.017	10	1.653 × 10^−3^	2.26	0.2238	ns
Pure error	2.920 × 10^−3^	4	7.300 × 10^−4^			
Sum	8.89	28				

Note: *p* < 0.01 is highly significant and denoted by **, *p* < 0.05 is significant and denoted by *, and *p* > 0.05 is not significant and denoted by ns.

## Data Availability

All relevant data from this study are included in this published article.
